# The pharmacy as a primary care provider

**DOI:** 10.3389/fpubh.2023.1221439

**Published:** 2023-08-24

**Authors:** Nachiket Mor, Dyuti Sen, Sarah Zaheen, Rubayat Khan, Priya Naik, Nayonika Basu

**Affiliations:** ^1^Banyan Academy of Leadership in Mental Health, Chennai, India; ^2^India Fellow, Independent Researcher, New Delhi, India; ^3^Jeeon Foundation, Dhaka, Bangladesh; ^4^Samhita Social Ventures, Mumbai, India

**Keywords:** primary care, health systems, informal providers, task shifting, community resource

## Abstract

**Introduction:**

Primary care is an essential component of any health system, but building high-quality primary care has proven to be a challenge for most developing countries. Among the multiplicity of providers in South Asia, one of the most ubiquitous channels through which not only medicines are obtained but also primary care advice is sought is the neighborhood pharmacy. There are widespread availability of pharmacies in South Asia. There is also good evidence that working with pharmacies in this way is a globally accepted idea, and there are several examples of countries, such as France and Nigeria, that have integrated pharmacies into their primary care systems and entrusted them with significant responsibilities.

**Methods:**

In this paper, we explore the potential of this channel as a formal primary care provider, with a particular focus on the South Asian context, by examining how pharmacies perform against the seven *Starfield attributes* of (i) first contact care, (ii) continuity of care, (iii) comprehensiveness, (iv) coordination, (v) family centredness, (vi) cultural competency, and (vii) community orientation. In the paper, we use data on pharmacies from four pharmacy-related interventions, one from Bangladesh and three from India, to carry out our analysis using the Qualitative Comparative Analysis (QCA) framework.

**Results:**

We find that even in the South Asian context, pharmacies provide several components of good primary care. As expected, they demonstrate a strong orientation toward the community in which they are located and are able to provide first-contact care. However, we find no direct evidence that they are able to offer continuity of care or bring to bear family-centredness and cultural competency when dealing with their patients. It is encouraging, however, that while there is no formal evidence of this in any of the interventions, multiple anecdotal examples suggest that pharmacists do indeed do much of this, but perhaps in an informal and inconsistent manner.

**Discussion:**

The evidence from these studies provides support for the view that pharmacies have many of the inherent characteristics needed to become an effective primary care channel and already play an important role in providing access to health information and care. However, it is also clear from the research that without additional training and access to tools, pharmacies will not have the competency or knowledge necessary to provide these services or even act as an effective gateway to other healthcare providers. To fully unlock this opportunity, therefore, any organization that wishes to engage with them will need to have the vision and patience to work with this network for an extended period of time and not merely aspire for incremental improvements but have a strategy in place that fundamentally changes the capabilities and the roles that pharmacies can play.

## 1. Introduction

Primary care is an essential component of any health system (1), but building high-quality primary care has proven to be a challenging proposition in many health systems. In India and Bangladesh, this challenge is further magnified, among other things, due to the severely underfunded nature of our government-owned health systems (2) and the very small size of our corporate and insurance sectors in healthcare, both of which have, in any case, avoided any serious involvement with primary care. However, there are a wide variety of private and non-profit primary care providers (3) that could potentially offer a path forward. Neighborhood pharmacies are one group of such providers that could be explored. Several South Asian studies have pointed to both their ubiquity and the range of services offered by them. Some of these studies are discussed below.

### 1.1. Pharmacies in South Asia

A survey in the Ujjain district of Madhya Pradesh in 2011 (4) found that there were 475 pharmacies in the district (28 per 100,000 population) with 369 in urban areas of the district (58.4 per 100,000 population) and 106 in the rural areas (8.4 per 100,000 population). While the urban pharmacies were tightly clustered within the urban areas, the rural pharmacies were spread fairly evenly across the district. Since the district has an area of 6,091 km^2^ and is approximately square-shaped, the 106 rural pharmacies were likely, on average, to be about 8 km from each other, with the median rural consumer having to walk about 4 km (about 1 h) to get to one, which constitutes a reasonable degree of accessibility. In terms of medicine availability, the study found that antibiotics such as amoxicillin and injectable cephalosporin were available in more than 80% pharmacies (without any differences in rural and urban areas), and the availability of injectable dexamethasone was as high as 88.7%, suggesting a good availability of basic and advanced medicines even in relatively remote areas. However, the study found that while pharmacies were adequately staffed in terms of the number of people, most of the staff (more than 88%) were not formally qualified as pharmacists and that the availability of refrigerators and backup power constituted a significant gap, particularly in rural pharmacies where only 20% had one.

A study of pharmacies in the city of Patna in Bihar by Daftary et al. (5) found that in a TB screening and referral intervention, of the 105 pharmacies that were recruited into the study, 81% “actively participated in the intervention” with the “rate of registration of symptomatic patients [being] 62 times higher in the intervention group compared with the control group” and “TB diagnosis […] 25 times higher.”

A more recent study by Kalita et al. in Odisha (6), which surveyed more than 30,000 people (living in more than 7,500 households) and 1,021 private pharmacies, found that more than 90% of the pharmacies, in addition to medicines, also offered medical advice and, at an average of 11.37 h a day, were open and staffed almost twice as long as the public sector primary care facility and solo private sector healthcare providers. The study also found that while hospitals (public and private) were the primary care location chosen by more than 50% of the patients, private pharmacies were the next most important primary care location, even though they were not free. They scored even higher than free government primary care providers. The study also found that while there were concerns related to the competency of providers (pharmacies and solo providers), which led patients to choose hospitals even for primary care, in aspects such as convenient hours and medicine availability, the pharmacy outperformed all other channels.

Chowdhury et al. (7), in their study of 302 customers of 76 pharmacies within Dhaka in Bangladesh, found that 90% of the customers “sought care from the study pharmacy as their first point of care.” “Ease of access to pharmacies (86%), lower cost (46%), availability of medicine (33%), knowing the medicine seller (20%), and convenient hours of operation (19%)” were some of the reasons they gave as to why they preferred this channel. And, in addition to over-the-counter medicines, 42% had been dispensed antibiotics by the pharmacies.

### 1.2. International experience

Internationally, as the review by the International Pharmaceutical Federation (8) shows, pharmacists have been encouraged to play an active role in the treatment, prevention, screening, and referral of non-communicable diseases (NCDs) due to their unique position in the community. The review also shows that the range of countries that formally allow a whole suite of NCD-related services includes both developed and developing countries (see the Appendix for more details). A 2013 report by the Grattan Institute on solutions to address general practitioner (GP) shortages in Australia also recommended the use of pharmacists in primary care to, among other things, “help manage chronic conditions (9).”

However, the degree to which pharmacies participate in specific components of NCD care varies greatly from country to country. Table 1, drawn from the larger review by the International Pharmaceutical Federation (8), presents a smaller group of countries that have been chosen to show the wide variation in the range of services offered. It can be seen from the table that French pharmacists even offer protocol-based prescriptions, while those in Iran and Argentina only carry out measurements of selected parameters.

**Table 1:** Pharmacy practice in selected large countries (8).

**Method → Country ↓**	**BP**	**BMI**	**CVD**	**Blood glucose**	**HbA1c**	**Medicine review**	**Disease monitoring**	**Protocol- based prescriptions**
Argentina	✓							
Brazil	✓	✓	✓	✓	✓	✓	✓	
France	✓	✓	✓	✓		✓	✓	✓
Britain	✓	✓	✓			✓	✓	
Nigeria	✓	✓	✓	✓	✓	✓	✓	
South Africa	✓	✓	✓	✓		✓	✓	
Indonesia	✓		✓	✓		✓	✓	
Iran				✓				

BP, Blood Pressure; BMI, Body-Mass Index; CVD, Cardiovascular Disease; HbA1c, a marker of average blood sugar level over 90–120 days.

In Nigeria, one of the countries listed in Table 1, a community pharmacy survey by Ihekoronye and Osemene (10) found that they offer a wide range of primary healthcare services to the communities they serve, including the supply of medicines to treat endemic diseases, disease prevention, and vaccination. They also report that blood glucose screening devices were the most widely adopted technology among community pharmacists in Nigeria.

It can be seen from Table 1 that the French go the furthest in their deployment of pharmacists in primary care. This expanded role of community pharmacies in France has its own set of challenges (11, 12). However, it evolved in response to the continued shortage of doctors, the urgent need for primary care, and, at 33 per 100,000, the remarkably high density of pharmacies in France. It is interesting to note that the current situation is almost exactly the same in most developing countries in all three aspects, particularly in South Asia.

Although the message from the literature is very positive and the promise of ubiquity that pharmacies hold out is highly attractive, it is not yet clear, particularly in the South Asian context, what formal roles pharmacies can play and how well-prepared they are for them. In this study, we analyse data from four South Asian pharmacy-related interventions in an attempt to better understand these issues.

## 2. Methods

The pharmacy can be thought of simply as a physical location at which multiple healthcare providers, such as doctors, nurses, and community health workers, can be co-located so that patients visiting any one service provider can have the benefit of all the others being available at the same location. There are many good examples of this, such as the CVS MinuteClinic (13) in the United States and the *Farmacias Similares* in Mexico (14). These are good models but have the very challenges of cost and availability of these other types of human resources that we hope to address through the pharmacy. In this study, we define pharmacies as physical locations where all services are provided directly by pharmacists and their assistants and explore, in the South Asian context, the extent to which these pharmacies are prepared to play a larger primary care role while continuing to perform their main task of filling medical prescriptions and dispense medicines.

High-quality primary care can be defined in many ways. In this paper, we use the framework provided by Starfield (15), which lists four core attributes, (i) first contact care, (ii) continuity of care, (iii) comprehensiveness, and (iv) coordination, and three derivative attributes, (v) family centeredness, (vi) cultural competency, and (vii) community orientation. Using these seven attributes, we analyse four South Asian interventions. Three of them are from India, and one is from Bangladesh.

**Figure 1:**
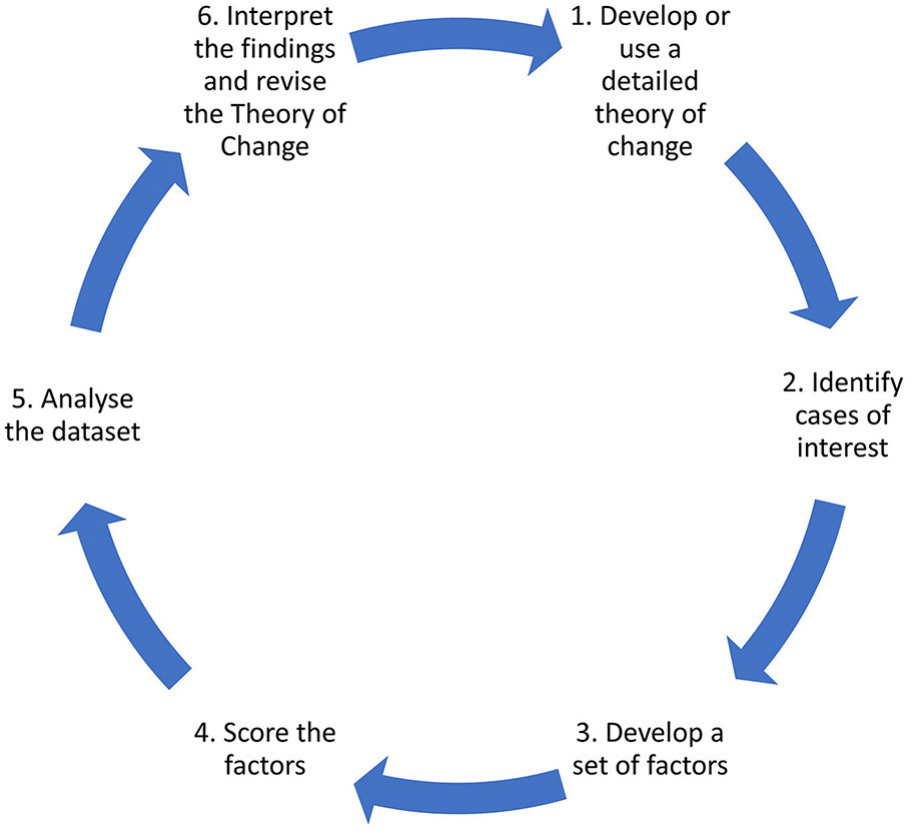
QCA steps [adapted from Simister and Scholz (17)].

In the study, using elements of the Qualitative Comparative Analysis (QCA) approach developed by Ragin (16), the analysis of these interventions is carried out by examining all the detailed quantitative and qualitative data available on them to determine which of the seven Starfield characteristics are present in each intervention. Since the sample size is small and the number of characteristics is larger, it is not possible to implement all the aspects of the QCA approach, such as “truth tables (16).” However, even with the partial application of the methodology, using the six-step approach suggested in Simister and Scholz (17), we believe that important insights can be gained from the analysis. The six steps are outlined in Figure 1.

This research is exempted from regulations for the protection of human subjects in research because it involves only analysis of information pre-collected for the purposes of health care operations of organizations from which data have been obtained. For the purposes of this research, the investigators received information in such a manner that the identity of the human subjects could not be ascertained directly or through identifiers linked to the subjects, the investigators have not contacted the subjects, and the investigators have not re-identified them (18).

## 3. Data

Each of the four interventions analyzed in this paper is described in brief below, with more details included in the Appendix.

### 3.1. Samhita pharmacy survey

Samhita is a social enterprise in India with a particular focus on corporate responsibility and harnessing the collective power of for-profit and non-profit entities to solve complex social problems at scale. Samhita partnered with the Cipla Foundation (associated with the Indian pharmaceutical company Cipla) to design the survey instrument. The intention was to capture an extensive set of data points about the pharmacists' profile, their attitudes toward their role within the community, business activities, business outcomes, and loan behaviors. The survey of 1,141 pharmacies was carried out in partnership with Cipla's field team, which supported the Samhita team during the data collection process. The results of the survey are analyzed in this paper. Two of the authors, Basu and Naik, are part of the Samhita team. Basu, as a member of the staff of Samhita, and Naik, as the Founder-CEO of Samhita.

### 3.2. Samhita pharmacy intervention

This was a healthcare intervention with 237 pharmacies in the suburbs of the Indian cities of Mumbai and Pune. The results of the intervention are analyzed in this paper. The intervention was supported in part by grants from the National Investment and Infrastructure Fund (NIIF). This is an organization promoted by the Government of India for the development of infrastructure. They did not play any role in any part of the design or implementation of the intervention. Through this intervention, in a 3-month period, the 237 intervention pharmacists were able to reach 2,845 unique customers. This resulted in 3,218 BMI [Body Mass Index = weight/height^2^ in *kgs*/*m*^2^)] and 2,930 blood pressure screenings. These data were made available to the researchers without any customer-level identifiers. More details are provided in the Appendix.

### 3.3. Jeeon pharmacy

This was an intervention in the rural areas of northern Bangladesh in which 42 pharmacies run by rural medical practitioners (RMPs) were onboarded onto a healthcare platform developed by Jeeon (19), and teleconsultations were provided to patients who came to these pharmacies. During the intervention period, the RMPs facilitated ~10,000 teleconsultations of 7,049 unique patients. Detailed data on these RMPs and these teleconsultations were available with Jeeon and were made available to the researchers without any customer-level identifiers. More details are provided in the Appendix. In the paper, we analyse these data. Two of the authors, Zaheen and Khan, were closely associated with the project. Zaheen, as the head of the Jeeon telemedicine unit, and Khan, as the Founder-CEO of Jeeon.

### 3.4. Jiyyo pharmacy

Jiyyo is an India-based social enterprise that, working with existing healthcare providers, plans to build a rural healthcare network across India using the name Jiyyo Mitra e-Clinics (20). Providers are connected to the Jiyyo app, which offers access to a network of doctors that they can use to provide enhanced services to their patients. Jiyyo has so far signed up more than 1,000 healthcare providers, including doctors, pharmacists and rural healthcare providers, in several states in the north and northeast of India. In this paper, we do a qualitative analysis of 19 interviews that were prerecorded by Jiyyo and made available on YouTube for public viewing.

The Jiyyo videos were independently analyzed by one of the authors, Sen. While doing the analysis, she was aware of her positionality as a public health professional and researcher with prior experience of working with pharmacists in rural India. She kept an open mind while analysing the videos and taking notes, to avoid the risk of interpreting the videos based on her own experience.

There was no engagement with the Jiyyo team for this paper. It is important to note that these videos were prepared by Jiyyo as part of their efforts to market their services and acquire more healthcare providers as their channel partners. This is very likely to have influenced their choice of partner to interview, the questions to ask, the decision to put these videos on YouTube, and any editing they may have done after recording the video.

There was no direct engagement of the authors while working on the paper either with pharmacists or their customers for any of the interventions. The analysis has been restricted to data that had already been collected by the organizations involved as part of their operations. However, as mentioned above, when reviewing the results, it is important to note that several of the authors were directly associated with the interventions analyzed in this paper.

### 3.5. Profile of pharmacists

The profiles of the pharmacists who participated in these interventions are given in Table 2 and discussed in the following paragraphs.

**Table 2:** Characteristics of pharmacists in the interventions.

**Intervention → Characteristic ↓**	**Samhita survey**	**Samhita intervention**	**Jeeon pharmacy**	**Jiyyo pharmacy**
**Sample size**				
Number of pharmacies	1,141	237	42	19
**Gender**				
Female	5%	22%	0%	0%
Male	95%	78%	100%	100%
**Age**				
< 30	8%	53%	0%	n/a^*a*^
30–50	74%	44%	76%	n/a^*a*^
>50	18%	3%	24%	n/a^*a*^
**Education**				
Pharmacy	16%	73%	0%	89%
College (non-pharmacy)	40%	23%	26%	0%
School/diploma	43%	4%	74%	0%
Other	2%	0%	0%	11%
**Services offered**				
Only medicines	57%	0%	5%	79%
Also advice	43%	100%	95%	21%

a n/a, not available.

#### 3.5.1. Age and gender profile

In three of the four interventions examined here, 95–100% of the pharmacists were male. Unusually, in the Samhita intervention, the number of female pharmacists was far higher at 22%. In terms of age, while there were pharmacists in some of the samples who were below the age of 30 years or above the age of 50 years, the majority were between the ages of 30–50 years, with, once again, the exception being the Samhita intervention where this number is somewhat lower at 44%, with a larger number (53%) being <30 years of age (Table 2).

#### 3.5.2. Education and experience

There were large variations between the samples on this dimension. In the Samhita and Jiyyo samples from India, the proportion of college graduates and even qualified pharmacists was over 75% (while Jiyyo had a very small sample, within it, more than 80% were formally qualified pharmacists). In the Jeeon sample, the group was much less formally educated (74% had just about finished their schooling), perhaps because the RMPs were all from rural areas. However, the pharmacists in all samples had a great deal of experience in their profession. While in the Samhita survey, only 43% said that they were offering medical advice, in the Jeeon sample, despite their comparatively lower educational levels, 95% offered pharmacy and healthcare services.

### 3.6. Intervention design

Specific interventions with pharmacists are described here (see the Appendix for more details).

#### 3.6.1. Technology and equipment

The Samhita intervention with 237 pharmacies provided their partners with BP monitors and weighing scales and access to an app that would allow them to record their readings and maintain patient history. The app was also the medium through which Samhita partners connected their customers with doctors. Jiyyo provided access to their telemedicine app to all their partners, along with training on how to use the app. Their partners could choose to upgrade to higher levels of offerings from Jiyyo, which offered more equipment. Jeeon offered its partners a suite of equipment, including an Android tablet (with the app), a printer (to print prescriptions), a BP machine, a glucose monitor, a weighing scale, a thermometer, and a pulse oximeter. Pharmacists were required to record these readings (some mandatory and some optional) and submit them as part of the patient's profile before the doctor reviewed the case and initiated the call.

#### 3.6.2. Training

In the Samhita intervention, pharmacists received a one-hour training in the use of a Blood Pressure (BP) measurement machine, weighing scales, the use of a digital platform to record BP and BMI measurements and how to communicate details about the problem of hypertension with their customers, such as its symptoms, possible treatments, and required lifestyle changes. Jeeon offered its network partners, the RMPs, a structured 2-day training program in the full SOAP (Subjective, Objective, Assessment, Plan) methodology (21)—the Appendix provides details of Jeeon's training program. In the Jeeon intervention, the team also took feedback on the pharmacist's performance from the doctors, to improve their training efforts. The limitations highlighted by the doctors included poor patient history taking such as unreliable vitals and symptoms, RMPs unable to communicate properly, aggressive behavior by RMPs, providing delayed or low-quality patient photographs, RMPs' unavailability during the scheduled call, and having insufficient phone balance. Jiyyo provided training to its partners in the use of its telemedicine app.

#### 3.6.3. Doctor network

This was a uniform feature of three interventions (excluding the Samhita survey). All the interventions offered access to a doctor network through the app that they gave to their pharmacy partners. It included access to a range of doctors who were available through the telemedicine platform included in the app. In the case of Samhita and Jiyyo, this was a video platform right from the beginning. The Jeeon intervention was, for the most part, in the form of phone-based consultation. The video was added only toward the end of the program in Jeeon once the rural network connectivity improved sufficiently in Bangladesh.

#### 3.6.4. Payment models

In all three interventions (excluding the Samhita survey), some form of payment was made to the pharmacy, either by the intervention or by the client/customer. In its intervention, Samhita offered a modest payment linked to performance to each pharmacist, but the customers were offered the screening for free. In both Jeeon and Jiyyo, the pharmacist charged each patient a modest sum per visit.

## 4. Results

As discussed above, each of the four interventions is carefully examined to determine which of the seven Starfield (15) characteristics pharmacists have. The ubiquitous presence of pharmacies in South Asia suggests that they are well-positioned to act as a first contact point for patients. And since they operate from a physical location within the pharmacy and their staff members are drawn from the local community itself, they could also address the requirements of continuity of care, family-centeredness, cultural competency, and community orientation. It is less clear, however, that without additional specialized training and access to larger integrated networks, they can provide comprehensive care or take on the responsibility of care coordination. Furthermore, although with this combination of capabilities, pharmacies may not be able to offer full-service primary care on their own, they could be well-positioned to address chronic diseases that require a high level of ongoing engagement with the patient. This engagement would be necessary to address issues such as the largely asymptomatic nature of many of these chronic diseases, making regular screenings essential. Engagement is also needed to address the enormous problem of non-adherence to treatment recommendations, which requires high levels of follow-up and strong cultural competencies, particularly when dealing with high-risk patients who are unwilling (or at times unable) to follow medical advice.

Based on this understanding of the potential primary care role of the pharmacy, in Table 3, we list the seven Starfield (15) characteristics and our initial hypotheses regarding those we expect to find. Each characteristic and its presence or absence in the pharmacies is analyzed in detail in the following paragraphs, and the results are summarized in Table 3.

**Table 3:** Potential pharmacy characteristics.

**#**	**Primary care characteristic**	**Potentially present in the pharmacy**
i	First contact care	Yes
ii	Continuity of care	Yes
iii	Comprehensive care	No
iv	Care coordination	No
v	Family centeredness	Yes
vi	Cultural competency	Yes
vii	Community orientation	Yes

### 4.1. First contact care

It is clear from the discussion that pharmacies in all settings offered a high level of accessibility and were often the first point of contact that the patient had with the healthcare system, even before the interventions. In the Samhita survey (see Appendix), 43% of the pharmacists said that they already offered medical advice to their consumers, and 51% felt that there was expressed demand from consumers for such services, particularly visible to them during the COVID-19 pandemic. In a short period of 3 months, the Samhita intervention, through its 237 pharmacists, was able to reach 2,845 unique customers and conduct 3,218 BMI and 2,930 blood pressure screenings (including repeats).

The Jeeon intervention enabled ~10,000 formal consultations for 7,049 unique customers, with 38% even coming in for repeat visits. On the Jiyyo platform, 89% of the pharmacists reported that the Jiyyo Mitra e-clinic will benefit patients as it will save them time and money in accessing quality healthcare. Seventy-four percent of them mentioned that due to the availability of telemedicine service in their own locality, patients in rural and remote areas will no longer have to travel to cities, state capitals, or other states to get treatment. Forty-two percent of the pharmacists felt that patients would benefit from better medical advice from qualified doctors and lower consultation fees. This quote from one of the Jiyyo pharmacists in Uttar Pradesh reinforces this point:

“During COVID, I saw patients coming to me and saying, give me medicine. I am ready to die, but I will not go to the hospital. Through the Jiyyo clinic, the patient does not have to go to the hospital, but rather the hospital comes to the patient, so this is a big help.” *Pharmacist, UP*

### 4.2. Continuity of care

Although none of the pharmacies showed any evidence of any form of proactive care or building risk-stratified cohorts so that they could track high-risk patients, given that they are based in the community and have customers coming in to see them for multiple purposes (including the purchase of non-medical consumer goods), it is possible to imagine that these pharmacies could ensure continuity of care since it is well-aligned with their own goals of ensuring repeat visits and more business from each customer. However, there was no direct evidence that they actually provided that kind of care to their customers in any of these four interventions. None of the interventions offered training to pharmacists on this aspect.

### 4.3. Comprehensive care

The Samhita intervention, through BP/BMI screenings followed by its teleconsultation facility, was designed to manage cardiovascular disease. Both the Jeeon and Jiyyo efforts were designed to offer support to their customers on a full range of healthcare conditions. The Jeeon team recognized that there were certain specialties, such as diabetes, hypertension, and dermatology, that worked much better with telemedicine and focused on them. Jeeon's training program trained and allowed its partners to comprehensively capture all the details about the patient and her condition before reporting them to the doctor. They found that pharmacists did not possess basic skills in areas such as palpation, capturing the pulse and breathing rate, and using the stethoscope, and offered them on-the-job coaching to get these to a minimum acceptable level of accuracy. The Jiyyo platform relied much more on having the pharmacist play the role of a concierge who would connect the patient to the doctor and simply dispense the prescribed medicine without directly getting into too much detail about the patient's health or performing any data collection.

### 4.4. Care coordination

Although 26% of the pharmacists on the Jiyyo platform reported that having multiple doctors with different specializations on one platform is an advantage, since no hospital has this facility, there was no direct evidence from them or from any of the other interventions that the pharmacists sought to coordinate care for their patients beyond the confines of the pharmacy itself. The Jeeon field team reported that while they did not track this in their intervention, they had indeed observed pharmacists providing care coordination services. During COVID-19, for example, they observed that 4,500 pharmacists in the city of Savar in Bangladesh, under the supervision of the sub-district health officer, played a crucial care coordination role, going as far as to accompany the patient to the referral hospitals to ensure compliance. It is important to note, however, that none of the interventions offered explicit training to pharmacists on this aspect.

### 4.5. Family centeredness

Despite the presumed belief (Table 3) that pharmacies, due to their continuous and longer-term presence in the community, would provide family-centered care, there was no direct evidence of this in any of the interventions. However, the Samhita team reported that their discussions with pharmacists, especially those who have been running a pharmacy for more than 15–20 years, suggest that they score high on cultural competence and family-centeredness because they have been serving the same community members for a prolonged period of time. They also tended to know details such as the medical history of the families they serve and the type of medicines they take on a regular basis. The Jeeon team also observed similar behavior from the pharmacists they worked with. However, it is important to note that none of the interventions offered explicit training to pharmacists on this aspect.

### 4.6. Cultural competency

There is a strong presumption of cultural competency because the pharmacists are from the same community and speak the same language as their customers. There was no direct evidence of its presence in any of the interventions, but the Jeeon team often observed that despite their general guidance that the doctor speaks directly to the patient, patients would often request pharmacists to speak on their behalf because they felt that they understood their situation better and could communicate it more accurately to the doctor.

### 4.7. Community orientation

Fifty-seven percent of the respondents in the Samhita survey saw themselves not just as business owners but as “supporters of people's health needs” and 25% as “members of the community.” Forty-two percent of the Jiyyo pharmacists felt that the patients will benefit from better medical advice from qualified doctors as well as lower consultation fees. During COVID-19, in Bangladesh, as in many other parts of the world, doctors and hospitals turned patients away, but according to a survey conducted by Jeeon, 95% of pharmacies remained open. When asked why, they said that it was their duty to support their communities during such a difficult time (if they wanted to continue operating as a trusted health provider in the future). They also said that they had limited choice in the matter as people would get them from their homes if the pharmacy was closed. This quote from one of the Jiyyo pharmacists reinforces their orientation toward the community:

“I am working here as a pharmacist for the last 11 years. I help patients with the right advice and provide them with medicines. Doctors are not there in our villages, so I joined the Jiyyo platform because, through this, I can support patients throughout the entire process, right from consulting with good doctors from big cities to providing them with the medicines as prescribed.” *Pharmacist, UP*

## 5. Discussion

As can be seen from Table 4, the evidence from the Samhita survey and the three interventions provides support for the view that pharmacies, even in the South Asian context, are providing several components of good primary care. As expected, they demonstrate a strong orientation toward the community in which they are located and are able to provide first-contact care. Judging by the number of consumers that they saw, the repeat consultations, and the number of screenings that were completed, it was also evident that customers trusted their pharmacists to act in their best interests. In terms of age, it can be seen from Table 2 that most are well below the age of 50 years, suggesting that they expect to remain in this profession for many years and, therefore, expect to benefit from making serious investments in building their capacities.

**Table 4:** Starfield characteristics present in each intervention.

**#**	**Intervention → Characteristic ↓**	**Samhita survey**	**Samhita intervention**	**Jeeon pharmacy**	**Jiyyo pharmacy**
1	First contact care	✓	✓	✓	✓
2	Continuity of care				
3	Comprehensive care	✓		✓	✓
4	Care coordination				
5	Family centeredness				
6	Cultural competency				
7	Community orientation	✓	✓	✓	✓

However, if we compare Table 4 with Table 3, we find that while many of the interventions do show that pharmacies aspire to provide comprehensive care, which is perhaps not their strength, there was no direct evidence that they were able to offer continuity of care or bring to bear family centeredness and cultural competency when dealing with their patients. These are critical capabilities and must be present if pharmacies are to represent an effective primary care channel for the control of chronic diseases in the South Asian, developing country, context. However, it is encouraging that, while there is no formal evidence of this in any of the interventions, the multiple examples mentioned above suggest that pharmacists are indeed doing much of this, but perhaps in an informal and inconsistent manner.

As can be seen from Table 2, the education and training of pharmacists is also an area of concern. Several are poorly trained, and it is not clear that even those with a formal pharmacy degree are being trained for the role they are actually performing (22), leave alone for a possible future in which they could be assigned increased healthcare responsibilities in a manner similar to the French pharmacists.

It can also be seen from Table 2 that an overwhelming majority of pharmacists are male. This limits their ability to engage closely with their female customers, particularly as it relates to chronic diseases. Jeeon's experience was that a majority of their patients were female and had a high degree of comfort with the male pharmacist. They even allowed the pharmacist to take, for example, pictures of their open breast scars/lumps to send to the doctor. However, despite this experience, there is no gainsaying that the presence of a female in the pharmacy would not only make it easier to perform some of these tasks even in highly conservative cultural contexts but also go further and, where necessary, even offer intimate procedures such as palpating the breast to check for early signs of cancer.

## 6. Conclusion

The evidence from these studies provides support for the view that pharmacies have many of the inherent characteristics needed to become an effective primary care channel and already play an important role in providing access to health information and care. In India, there are estimated to be over 800,000 pharmacies (23) or over 60 per 100,000 population, almost double the number in France (12). In Bangladesh, for a population of about 169 million (24) there are estimated to be a total of about 185,000 RMPs (25), indicating the presence of 109 RMP-pharmacies per 100,000 population, more than double the number in India. This suggests a very high and dense presence, with more than one pharmacy on average for every 2,000 population in India and over twice as many in Bangladesh. Given the eagerness demonstrated by the pharmacists in the above interventions to offer an expanded range of primary care services, the positive experiences that several countries, including, as discussed above, France and Nigeria, and the urgent need in developing countries like India to improve access to primary care, it would be critical to recognize the important role that pharmacists can play.

It is also clear from the study that pharmacies are already more than just mechanical dispensers of medicines in the South Asian context. And, given the futility and difficulty of trying to prevent pharmacists from going beyond mere the sales of medicines, and the critical need for this type of care in many parts of South Asia (and the developing world in general), it is instead important to explore, as many countries (see Table 1) have done, how protocols and training could be used to both improve the quality of clinical services provided by pharmacies and even encourage them to, as it were, step up to this role. Given the density of pharmacies in the South Asian context, the trust and respect they enjoy, and unlike traditional healers, the close connection pharmacies have with mainstream medicine, they could be particularly well-suited to address the challenge of the rapidly increasing burden of chronic diseases (8).

However, for this to happen, it is necessary to shift the Nash Equilibrium (26) from the current situation in which the pharmacies in South Asia are indeed providing a range of services but at a level far below what they could do, to one in which they are able to realize their full potential. As the above-mentioned COVID-19 example from the city of Savar in Bangladesh illustrates, these healthcare providers can be real assets for those willing to work with them. A disease surveillance program Jeeon ran also showed that pharmacists can be used effectively to capture health data and conduct surveillance at scale at a relatively low cost. This type of data can be a useful by-product of a digital app that they use to treat and/or manage their patients.

In terms of regulation, the telemedicine guidelines in India (27) have, for example, made it possible for doctors to operate remotely thus enabling pharmacists to truly become a steward of the health of their customers, bringing in the doctor as and when required by law, thus removing any fundamental regulatory barrier. This opens an opportunity for private and public sector organizations to engage the fragmented pharmacy sector and build highly customer-centric networks.

However, to unlock this opportunity, any organization that wishes to engage with them will need to have the vision and the patience to work with this network for an extended period of time and not merely aspire for incremental improvements, but have a strategy in place that fundamentally changes the capabilities of pharmacists and the roles that they can play and transforms the provision of healthcare, using that network. Given all the pressures that in-person pharmacies currently face from e-Commerce players, this is the right time to engage them. Earlier, they may have been more resistant to change, but if we wait any longer, the risk is that these pharmacies may start to disappear entirely.

## 7. Limitations

The principal limitation of the study is the small number of cases analyzed. However, the rich context of these cases provides deeper insight into the underlying issues that large-sample regression analysis is incapable of. The other limitation is that since the data have been obtained from the operational records of these cases, it is not possible to explore how these different pharmacies react to different intervention designs. However, the design of such interventions would have to carefully navigate deep ethical and regulatory concerns, which are absent when using operational data.

An added limitation is that the data reviewed come from pharmacies that are already participating in some type of established network or social enterprise that provides them with access to additional training, equipment, and telemedicine networks, which differentiates them from a typical community pharmacy.

## Data availability statement

The original contributions presented in the study are included in the article/Supplementary material, further inquiries can be directed to the corresponding author.

## Author contributions

NM conceptualized and designed the entire study, analyzed the data, and wrote the first draft of the manuscript and the attached data Appendix. DS, SZ, RK, PN, and NB provided parts of the data sets needed for the study, participated closely in analyzing these data sets, and drafting portions of the manuscript. All authors reviewed the results and approved the final version of the manuscript.
