# Supplementary material for: The pharmacy as a primary care provider
Source: Front Public Health. 2023 Aug 24;11:1221439. doi: 10.3389/fpubh.2023.1221439 (PMC10483221; doi:10.3389/fpubh.2023.1221439)
Supplement: Supplementary file 1 [file Data_Sheet_1.PDF]

The Pharmacy as a Primary Care Provider

Nachiket Mor[1], Dyuti Sen[2], Sarah Zaheen[3], Rubayat Khan[3], Priya Naik[4], and Nayonika Basu[4]

[1]Banyan Academy of Leadership in Mental Health, Chennai, India
[2]India Fellow, New Delhi, India
[3]Jeeon Foundation, Dhaka, Bangladesh
[4]Samhita Social Ventures, Mumbai, India

May 12, 2023

A International Experience

Internationally, as the review by the International Pharmaceutical Federation (1) clearly brings out, pharmacists have been encouraged to play an active role in the treatment, prevention, screening, and referral of non-communicable diseases (NCDs) because of their unique position in the community. It can be seen from table 1 that the range of countries that formally permit a whole suite of NCD-related services includes both developed and developing countries. However, the extent to which pharmacies participate in specific components of NCD care varies greatly from country to country (table 2), with French pharmacists offering even protocol-based prescriptions while those in Iran and Argentina only carrying out measurements of selected parameters.

Method	Countries & territories where the service is offered
BP (Blood Pressure) Measurement (Hypertension)	Albania, Argentina, Armenia, Australia, Austria, Benin, Brazil, Cabo Verde, Cameroon, Canada, China, China Taiwan, Costa Rica, Croatia, Czech Republic, Ecuador, Fiji, Finland, France, Germany, Ghana, Great Britain, Hungary, Indonesia, Ireland, Israel, Jordan, Lebanon, Mali, Malta, Mauritius, Nepal, Netherlands, Nigeria, Pakistan, Panama, Philippines, Portugal, Romania, Sierra Leone, Singapore, South Africa, Spain, Sweden, Tanzania, Turkey, Uruguay, USA, Zimbabwe
BMI (Body Mass Index) Determination (Obesity)	Albania, Australia, Austria, Brazil, Cameroon, China, China Taiwan, Croatia, Czech Republic, Ecuador, Fiji, France, Germany, Ghana, Great Britain, Hungary, Ireland, Israel, Lebanon, Malta, Mauritius, Nepal, Netherlands, Nigeria, Pakistan, Portugal, Sierra Leone, Singapore, South Africa, Spain, Sweden, Tanzania, Turkey, Uruguay, Zimbabwe
CVD (Cardiovascular Disease) Risk Assessment Questionnaire (smoking habits, physical activity, diet)	Australia, Brazil, Cameroon, Canada, Croatia, Czech Republic, Ecuador, France, Great Britain, Hungary, Indonesia, Ireland, Israel, Mauritius, Netherlands, Nigeria, Panama, Portugal, Singapore, Spain, Sweden, Tanzania, USA Point-of-care tests for total blood cholesterol Austria, Brazil, China, Costa Rica, Croatia, Ecuador, Fiji, Germany, Hungary, Ireland, Israel, Lebanon, Malta, Netherlands, Nigeria, Portugal, South Africa, Spain, Sweden, Tanzania, USA

Method	Countries & territories where the service is offered
Blood Glucose (Glycaemia)	Albania, Armenia, Australia, Austria, Brazil, Cabo Verde, Cameroon, China, China Taiwan, Costa Rica, Croatia, Czech Republic, Ecuador, Fiji, France, Germany, Ghana, Hungary, Indonesia, Iran, Ireland, Israel, Jordan, Lebanon, Malta, Mauritius, Nepal, Netherlands, Nigeria, Oman, Pakistan, Panama, Philippines, Portugal, Romania, Sierra Leone, South, Africa, Spain, Sweden, Tanzania, Uruguay, USA, Zimbabwe
HbA1c (Glycated Haemoglobin A1c) for Diabetes	Australia, Brazil, Mauritius, Oman, Pakistan, Panama, Portugal, Romania, Spain, Sweden, USA Diabetes risk assessment questionnaire Australia, Austria, Belgium, Canada, Finland, Germany, Hungary, Mauritius, Nigeria, Portugal, Singapore, Spain, Sweden, Tanzania, Turkey
Medicines use review	Afghanistan, Australia, Benin, Brazil, Cameroon, Canada, China Taiwan, Costa Rica, Croatia, Czech Republic, Ecuador, Finland, France, Great Britain, Hungary, Indonesia, Israel, Kenya, Lebanon, Malta, Nepal, Netherlands, New Zealand, Nigeria, Pakistan, Panama, Paraguay, Portugal, Romania, Rwanda, Singapore, Slovenia, South Africa, Spain, Sweden, Switzerland, Tanzania, Turkey, USA
Disease management (monitoring)	Australia, Belgium, Brazil, Cameroon, Canada, China, Taiwan, Costa Rica, Croatia, Czech Republic, Ethiopia, France, Great Britain, Hungary, Indonesia, Malta, New Zealand, Nigeria, Panama, Paraguay, Portugal, Romania, Singapore, Slovenia, South Africa, Spain, Sweden, Turkey, USA Renewal of prescriptions based on protocols Afghanistan, Benin, Canada, Czech Republic, France, Lebanon, Malta, Mauritius, Netherlands, Pakistan, Slovenia, Spain, Sweden, Tanzania, USA, Zimbabwe
Renewal of prescriptions based on protocols	Afghanistan, Benin, Canada, Czech Republic, France, Lebanon, Malta, Mauritius, Netherlands, Pakistan, Slovenia, Spain, Sweden, Tanzania, USA, Zimbabwe

Table 1: Pharmacy services (1)

Country/Method	BP	BMI	CVD	Glucose	HbA1c	Medicine Review	Disease Monitoring	Protocol-based Prescriptions
Argentina	✓							
Brazil	✓	✓	✓	✓	✓	✓	✓	
France	✓	✓	✓	✓		✓	✓	✓
Britain	✓	✓	✓			✓	✓	
Nigeria	✓	✓	✓	✓	✓	✓	✓	
South Africa	✓	✓	✓	✓		✓	✓	
Indonesia	✓		✓	✓		✓	✓	
Iran				✓				

Table 2: Pharmacy practice in selected large countries (adapted from table 1)

Zone/City	Tier 1	Tier 2	Tier 3	Tier 4	Tier 5	Tier 6	Total	%
North							48	6%
South							193	25%
East							207	27%
West							227	30%
Total	39	219	297	78	117	13	1141	
%	5%	29%	39%	10%	15%	2%		100%

Table 3: Samhita Survey: Cities & Zones

Gender/Age (years)	< 25	25-35	35-45	45-55	≥ 55	Total	%
Female	3	12	29	11	3	58	5%
Male	15	127	438	373	129	1082	95%
Other						1	0%
Total[a]	18	139	467	384	132	1141	
%	1.6%	12.2%	41.0%	33.7%	11.6%		100%

Table 4: Samhita Survey: Age & Gender

Note:[a]The data needed in the main paper for the age distribution are $\leq 30 = 95$ (8.3%); 30-50 = 837 (73.4%); $> 50 = 209$ (18.3%) out of a total of 1141 (100%)

B Samhita Pharmacy Project

This project is an intervention by Samhita (the organisation with which two of the authors, Basu and Naik, are associated) in which 237 pharmacies across the suburban areas of two large cities in India, Mumbai and Pune, were partnered to provide enhanced health care services. This intervention was preceded by a survey of 1141 pharmacies across the country. In this note, the survey and the intervention are both discussed in some detail.

B.1 Samhita Survey

For the survey, Samhita reached out to 1141 pharmacists across the country. It can be seen from table 3 that approximately 70% of these pharmacies were located in Tier 2 & Tier 3 cities. From table 4, it can be seen that 95% of these pharmacists were male. About 40% (table 4) of them were in the 35-45 years age group, suggesting that they are likely to have the ability to stay with this business for the next several decades. While only about 16% had pharmacy degrees, more than 90% of them were either college graduates or diploma holders (table 5) – indicative of an enhanced ability to absorb and work with medical protocols and procedures. While 91% managed with only one staff member (table 6), 88% of them had more than 50 customers come into their establishments with 61% of them seeing anywhere between 100 to 500 customers per day (table 7). It can also be seen that 43% of them were already providing some form of medical advice to their customers (table 8), 57% saw themselves as supporters of the health needs of people (table 9), and during COVID-19 51% have already experienced an increased demand from consumers for medical advice (table 10).

Level	Number	%
M.Pharm	5	0%
B. Pharm	184	16%
College degree (non-medical)	450	40%
12th pass (10+2)	54	5%
Below 12th class	24	2%
Diploma	400	36%
Don't know	1	0%
Other	7	1%
Refused to answer	16	1%
Total	1141	100%

Table 5: Samhita Survey: Education

#/ Pharmacy	Number of Pharmacies	%
1	1037	91%
2	70	6%
3	20	2%
4	2	0%
5	2	0%
6	1	0%
7	1	0%
9	2	0%
25	1	0%
50	1	0%
Total	1141	100%

Table 6: Samhita Survey: Number of Pharmacists in each Pharmacy

Range	Number	%
< 50	137	12%
50-100	92	8%
100-500	691	61%
500-1000	189	17%
≥ 1000	32	3%
Total	1141	100%

Table 7: Samhita Survey: Daily Volume of Business

Services Provided by the Pharmacist	Number (out of 1141)	%
Medicines for various illnesses	1017	90%
Medical Guidance	479	43%
FMCG Products	563	50%
Refused to Answer	16	1%

Table 8: Samhita Survey: Services

Pharmacists Self-Image	Number (out of 1141)	%
Member of medical community	508	45%
Supporter of people's health needs	649	57%
Business owner	818	72%
Member of the community	286	25%
Householder trying to earn an income	163	14%

Table 9: Samhita Survey: Self-Image of Pharmacist

Impact of COVID-19 on the Pharmacy	Number (out of 1141)	%
Increase in profits	289	25%
Increase in numbers of customers	333	29%
Customers are seeking more advice	580	51%
Decrease in staff availability	272	24%
Decrease in access to products	212	19%
Business was down	128	11%

Table 10: Samhita Survey: Impact of COVID-19

Characteristic	Number[a]	%
**Gender**		
Female	52	22%
Male	185	78%
**Age**		
< 30	125[b]	53%
30-50	103[b]	44%
> 50	6[b]	3%
**Education**		
Pharmacy	172	73%
College (non-pharmacy)	55	23%
School/Diploma	10	4%
Other	0	0%
**Services Offered**		
Only Medicines	0	0%
Also Advice	237	100%
**Employee #**		
0	3	1%
1	56	24%
2	84	35%
3	57	24%
4	26	11%
≥ 5	11	5%

Table 11: Samhita Intervention Pharmacy Characteristics
Note:
[a]all the numbers in the column are out of a total sample size of 237 pharmacies.
[b]age was not available in the data for 3 pharmacies, so these numbers are out of a total of 234.

B.2 Samhita Intervention

Program Component	Description
Equipment	Distribution of digital Blood Pressure Monitors and weighing scales to pharmacists. The weighing scale, along with the height, would help in computing the Body Mass Index (or BMI=kg/m^2) to classify a person into underweight (BMI < 18.5), normal weight ($18.5 \leq$ BMI ≤ 23.5), overweight (($23.5 <$ BMI ≤ 25.0) and obese (BMI > 25.0) categories.
Technology	Pharmacists were trained in observing and recording the readings of blood pressure and BMI in an app for customers.

Program Component	Description
Training	Pharmacist was also given some basic awareness training on communicating awareness of Hypertension, its symptoms, possible lines of treatment and associated lifestyle changes required.
Enabling screening	Pharmacists in the intervention to offer free BP and BMI screening to the walk-in customers at the pharmacy.
Doctor network	Pharmacists were provided with the necessary connections to provide a point of access to the customer to a network of doctors for availing teleconsultation should they desire, for the purpose of enablement of a care continuum.
Incentives	Pharmacists were incentivized on a pay-for-performance model for the screenings and teleconsultations happening through them.

Table 12: Intervention Components

Subsequent to the survey, a program was launched with 237 pharmacies across the suburban areas of Mumbai and Pune. The program aimed to empower pharmacists with the knowledge, tools, resources, and incentives to become trusted healthcare providers (beyond selling medicines) to their local and possibly underserved communities. Through this program, pharmacists supported citizens with easy, quick, and free-of-cost access to Blood Pressure (BP) and Body Mass Index (BMI) screening that correlates to two of the most common lifestyle diseases - Hypertension and Obesity. The summary characteristics of these pharmacies are listed in table 11, and the key intervention components are listed in table 12.

Through this intervention, in a period of 3 months which included the sensitization of the pharmacists, 237 pharmacists were able to reach out to 2845 unique customers. This resulted in 3218 BMI screenings and 2930 blood pressure screenings.

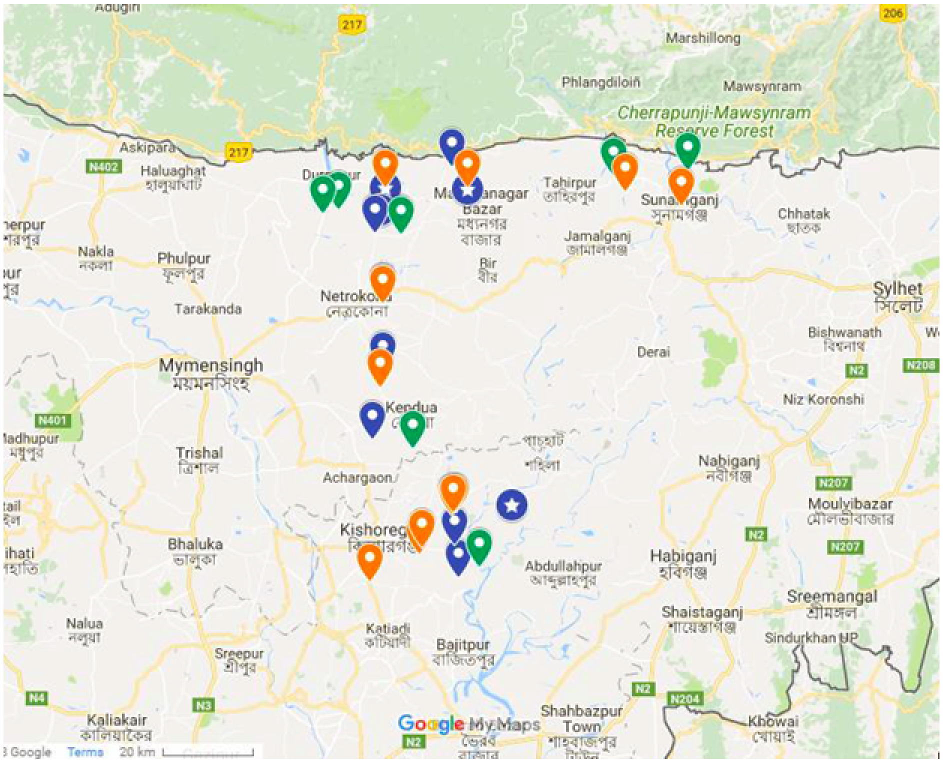

Figure 1: Jeeon Pharmacy Network in Northern Bangladesh (36 of 42)

C Jeeon Pharmacy

Jeeon started its operation in 2015 with the vision of using rural informal healthcare providers as a means to deliver healthcare services to the rural population in Bangladesh. They developed a telemedicine platform on which they onboarded 42 rural medical practitioners (RMPs). Through the platform, they conducted over 10,000 teleconsultations in 4 subdistricts across northern Bangladesh (figure 1). The RMP or the "village doctor" is a category of health provider that, despite not being recognised by the government, serves as a first point of contact for over 65% of the rural population because they are seen as a trusted source of healthcare by the community they operate in (2).

Evidence suggests that there are over 185,000 RMPs (3) covering every corner of the country. They usually have about 10-12 years of formal schooling and complete some unaccredited or short courses (up to 6 months) related to their medical practice. A small fraction complete paramedical certification courses up to 3 years in duration. These training courses usually cover basic anatomy and physiology, pharmacology, microbiology and pathology, first-aid, and family planning. The minimum qualification for enrolling in RMP-related courses is a secondary school certificate (grade 10 completion) or equivalent.

They are also legally required to get a Grade-C pharmacy certification and a drug license if they want to operate a pharmacy. However, most RMPs own pharmacies informally without fulfilling these requirements. Before onboarding them onto the Jeeon Telemedicine platform, RMPs were selected based on the criteria given in table 13. After meeting the minimum eligibility criteria, the RMPs had to undergo 3 days of basic training in-house, where they

Criterion	Acceptable Range
Age (in years)	30-45
Length of presence in the marketplace	> 5 years
Daily patient flow	> 10
Education	> 12 years of schooling
Shop size and location in the marketplace	Medium to large, centrally located
Current medicine inventory	Reasonable
Experience using a smartphone	Basic
Ability to invest	Yes

Table 13: Jeeon Project: Pharmacist Selection Criteria

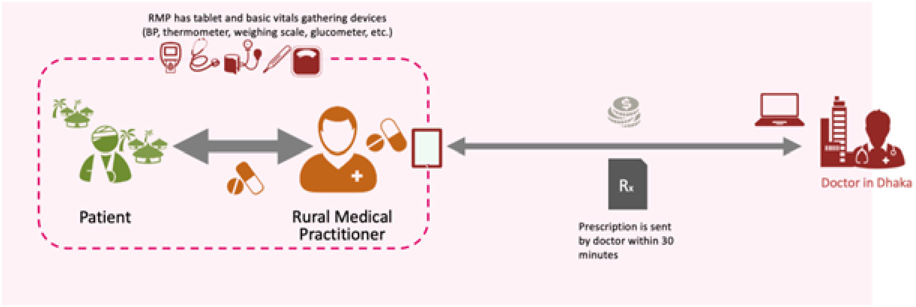

Figure 2: Jeeon Telemedicine Model

would be trained on patient triage, patient counselling, data entry, rapid diagnostics test kits and use of Jeeon's technology (table 14).

After the training, the RMPs were expected to act as telemedicine assistants for the remote doctors through the Jeeon platform, gathering required patient data and vitals and also facilitating the conversation between the patient and doctor (figure 2). Due to bandwidth limitations during the initial period, the intervention included phone calls as a way of communication and only during the last few months of the project, when 3G connectivity was established, was video calling integrated. When a patient arrives at the Jeeon service point for consultation, the Jeeon RMP registers her as a new patient, creates an ID card which is unique for each patient and books a slot from the existing doctor slots available on the platform (figure 3). The RMP then proceeds to collect the patient's vitals, conducts a series of physical examinations (figure 4), and collects the patient's medical history, which includes chief complaints, drug history, personal history, and family history. Whenever applicable, they send pictures of visibly discernible conditions and test results. If required, the RMP also conducts further tests (such as blood glucose levels, blood tests, and urine tests) depending upon the availability of the test at the service point. All this data is then entered into the Jeeon platform on the tablet and shared with the doctor.

Day	Activities	Time Assigned (hh:mm)
Day 1	**Medical Training**	08:30
	Introduction (learning objectives, time allocation, etc.)	00:15
	Watching Videos + Quick demonstration & review after each video	01:45
	Break	00:10
	Hands-on practice of each activity/examination.<br>(In pairs – Instructor to rotate across participants for each exam)	02:30
	Break for lunch and prayers	01:00
	Explanation and use of the forms & checklists	00:20
	Take participant through entire experience+quick feedback	00:65
	Break	00:10
	Take participant through entire experience+quick feedback	00:45
	Card game	00:20
	Conclusion	00:10
Day 2	**Technology Training**	08:30
	Introduction (learning objectives, time allocation, etc.)	00:10
	Basics of the Tablet (hands-on)	01:30
	Basics of the Printer (hands-on)	00:15
	Break	00:15
	Registering a patient	00:20
	Sending an appointment	00:30
	Prescription download & print	00:15
	Account refill	00:30
	How to take a good picture (video) + Review	00:30
	Break for lunch and prayers	01:00
	Taking a picture (hands-on practice)	00:30
	Sending extra pictures	00:20
	Break	00:15
	Full process practice	02:00
	Conclusion	00:10
Day 3	**Detailed Review**	08:30

Table 14: Jeeon Project: Pharmacist Training Program

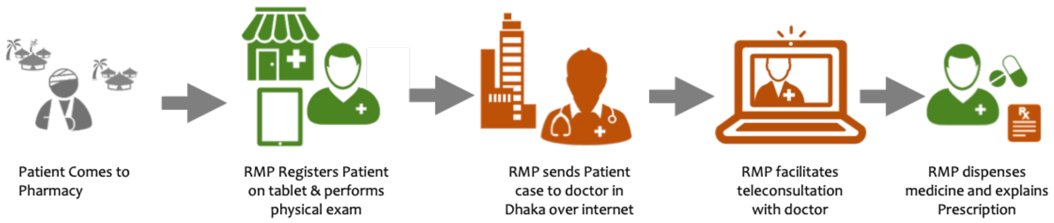

Figure 3: Jeeon Consultation Process

Once the doctor reviews all the information sent by the RMP, the doctor calls back the RMP over the phone (and later video), at which point the RMP intermediates a conversation between the doctor and the patient. The RMP is expected to provide a clear and complete history of the patient and his/her condition and help the doctor probe deeper, where necessary. Once the RMP presents the patient's case to the doctor, the doctor then directly talks to the patient via audio/video conference call facilitated by the RMP. The Doctor explains the treatment plan to the patient and the RMP. Once the call is concluded, the doctor types the prescription and sends it over to the RMP via the Internet. Upon receiving the prescription on the tab, the RMP prints it out and explains the prescription, with special emphasis on the medicine section, so that the patient fully understands how to take the prescribed medication (times of day and any special instructions). The RMP also thoroughly explains the advice in the prescription and encourages the patient to do the investigations prescribed by the doctor. The RMP usually charges Tk 300 for new patient consultations and Tk 50 for follow-up visits.

All 42 pharmacies working on the Jeeon platform were located in middle to large-scale markets in rural areas, which are frequently visited by patients from neighbouring villages. All the RMPs were male, and a majority of them, 76%, were in the age group 30-49 years (table 15). The educational qualification of the pharmacists differed, with 74% having completed at least high school, 24% having a Bachelor's degree, and 3% having a Master's degree (table 15). None of them had a degree in pharmacy, but all of them had undertaken the Local Medical Assistant and Family Welfare (LMAF) course, a short medical training course for RMPs. The LMAF certification, while conferring a measure of credibility to the RMP, does not have formal government accreditation and is provided by training institutes of inconsistent quality.

In addition to these formal qualifications, 7% of the pharmacists had had 3 months of prior training, 53% 6 months, 38% 1 year, and 3% 2 years (table 15). 38% of RMPs had 0-10 years of experience in the field, 43% 11-20 years, 17% 21-20 years, and 3% had more than 30 years of experience (table 15). The average length of experience was 14 years. Almost all, i.e., 95%, were both pharmacy owners and RMPs, and 5% were only RMPs and did not own a pharmacy (table 15). As per their reported annual income, 36% were categorized as small-scale operators, 43% as medium-scale operators and 1% as large-scale operators (table 15).

The RMPs facilitated approximately 10,000 teleconsultations of 7049 unique patients throughout the period. Out of them, 62% (n=4361) patients made one visit for consultation and 38% (n=2688) made 2 or more visits. The median volume of patient consultations

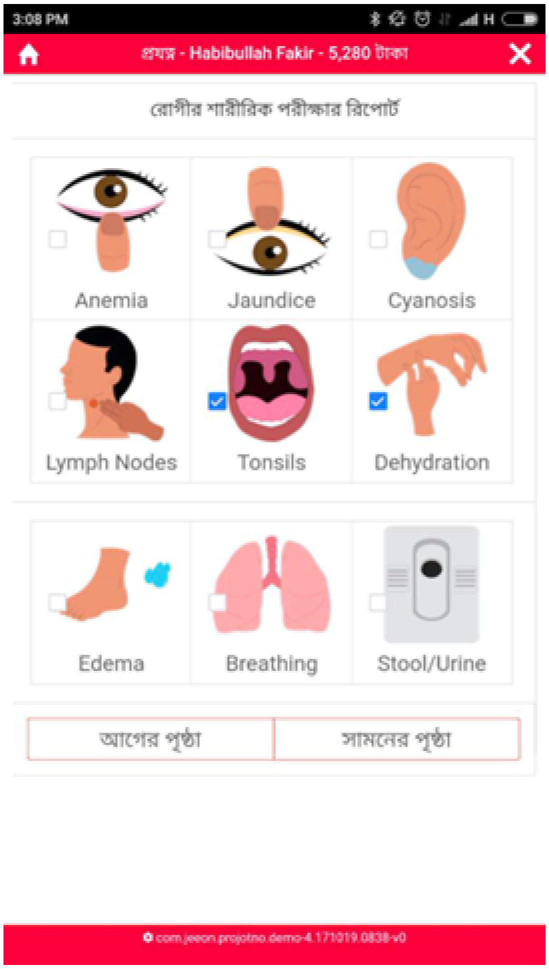

Figure 4: Pre-consultation Examination by the Jeeon Pharmacist

Characteristic	Number	%
Age		
30-49 years	32	76%
$\geq$ 50 years	10	24%
Education		
SSC (Class 10)	16	38%
HSC (Class 12)	15	36%
BA	10	24%
MA	1	3%
Years of prior training		
3 months	3	7%
6 months	22	52%
1 year	16	38%
2 years	1	3%
Years of experience		
0-10	16	38%
11-20	18	43%
21-30	7	17%
31 and above	1	3%
Scale of operation		
Small ($\leq$ Tk 1 lakh)	15	36%
Medium (Tk 1 lakh-Tk 3 lakh)	18	43%
Large ($\geq$ Tk 3 lakh)	6	14%
Data not available	3	7%
Service Type		
Pharmacy & consultation	40	95%
Consultation only	2	5%

Table 15: Jeeon Pharmacy Participant Characteristics (n=42)

(non-unique patients) facilitated by 41 RMPs was 189 [IQR=88,363]. While 31% facilitated between 3-103 patient consultations (n=13), only one facilitated more than 1000 consultations. The RMPs were associated with Jeeon for different durations, 31% (n=13) were associated for a year, 26% (n=11) for two years, 31% (n=13) for four years and 12% (n=5) for five years.

D Jiyyo Pharmacy

Jiyyo Mitra e-clinic is a telemedicine initiative in India connecting patients from rural and semi-urban areas with qualified doctors from institutions like AIIMS, PGI Chandigarh and SMS Jaipur. The telemedicine operators who run the e-clinics have a medical background and are either doctors, pharmacists or rural healthcare workers. Their role is to initially screen patients, understand their illness and then connect them with appropriate doctors from the Jiyyo panel. Since 2020, Jiyyo has set up over 1000 e-clinics in 80 districts of Uttar Pradesh, Rajasthan, Madhya Pradesh, Bihar, Jharkhand, Punjab, Haryana, Uttarakhand and Assam, providing service to more than 100,000 rural patients.

A qualitative study was undertaken to understand the perception of pharmacists towards the e-clinic and telemedicine model. Secondary data was collected from Jiyyo's YouTube channel, which included 96 video testimonials from all the Jiyyo Mitra e-clinic operators. As a first step, all videos were watched to identify the videos with pharmacists as operators. The videos were approximately 2-5 minutes long and recorded in Hindi. In the second step, the 19 videos of pharmacists were re-watched, and detailed notes were taken in English for in-depth analysis. All notes were taken and saved on a Microsoft Excel 2021 file. The data analysis was done manually, and each video note was reviewed line by line. An inductive approach was followed for coding, charting and interpretation. The codes were then combined into broad themes, and the final themes were generated after re-reading all the notes.

Out of the 19 pharmacists, 12 were from Uttar Pradesh, 3 from Rajasthan and Jharkhand each and 1 from Haryana. Table 16 provides an overview of the participants' characteristics. In all videos, the Jiyyo staff, as well as the health partners, were male. In 18 videos, a Jiyyo associate introduced the Jiyyo Mitra e-clinic and then facilitated the brief interview and discussion with the pharmacists, and in one instance, the pharmacist self-recorded the video. All videos were recorded in the health facility itself. The questions included asking the pharmacist to introduce themselves, how they felt being a Jiyyo partner, how they got to know about Jiyyo, why they have joined the Jiyyo Mitra e-clinic platform, how they think telemedicine will be useful in their context, any patient testimonials, message for other health partners, and how they feel about the support from Jiyyo team. The questions were not asked consistently in all videos. Two key themes were identified in the Jiyyo pharmacy videos: (i) how the pharmacists felt about the telemedicine initiative, and (ii) what possibilities are there for pharmacies as a channel in the future. The analysis of the videos and a few selected quotes are captured in table 17. In this study, most of the pharmacists reported the benefits of telemedicine for patients and communities, especially in rural contexts.

D.1 Reflexivity concerns

All the video interviews were analysed independently by one of the authors of the paper (Sen). She recognises her positionality as a public health professional and researcher with prior experience of working with pharmacists in rural India. She kept an open mind while analysing the videos and taking notes to avoid the risk of interpreting the videos based on her own experience. These videos were prepared by Jiyyo as a part of their efforts to market their services and acquire more healthcare providers as their channel partners. This is very likely to have influenced their choice of partner to interview, the questions to ask,

Characteristic	Participants (n=19)	%
Gender		
Male	19	100%
Female	0	0%
Qualifications		
Pharmacist (B Pharma)	14	74%
Pharmacist (D Pharma)	2	11%
Pharmacist & GNM	1	5%
Not clear	2	11%
Health Facility		
Pharmacy	15	79%
Pharmacy & clinic	4	21%

Table 16: Jiyyo Pharmacy Participant Characteristics

the decision to put these videos on YouTube, and any editing that they may have done after recording the video.

Analysis	Quote
**How the pharmacists felt about the telemedicine initiative**	
17 pharmacists reported that the Jiyyo Mitra e-clinic will benefit patients as it will save their time and cost in accessing quality healthcare. 14 of them mentioned that due to telemedicine service being available in their own locality, patients in rural and remote areas will no longer need to travel to cities, state capitals or other states to get treatment. 8 pharmacists felt that the patients will benefit from both better medical advice from qualified doctors as well as lower consultation fees.	"Our village is 50kms away from the city, patients have to travel to nearby cities or even Delhi, Lucknow to consult doctors and their rates are also high, so it's not affordable for all patients. Jiyyo set-up will help the common man. Patients who consulted doctors here are happy, and it saves them money and time." *Pharmacist, Haryana*
Another possible benefit to patients due to the Jiyyo setup, as highlighted by 7 pharmacists, was the choice to avoid going through the hassle of going to a hospital far away from their homes.	"During covid, I saw patients coming to me and saying, give me medicine. I am ready to die but will not go to the hospital. Through Jiyyo clinic, the patient does not have to go to the hospital, but rather the hospital comes to the patient, so this is a big help" *Pharmacist, UP*

Analysis	Quote
The pharmacists mentioned that since the pandemic, many doctors have already started telemedicine, and 5 of the pharmacists felt that telemedicine or online consultation is the future of medical care. 5 pharmacists also reported that having multiple doctors with different specialisations under one platform is an advantage, as no hospital has that facility. One pharmacist each felt that through the telemedicine platform, their own knowledge as pharmacists will improve, and their practice will grow over time as they will attract more patients.	"A message for other pharmacists would be to join this platform because our knowledge as pharmacists will also improve through this, and patients will benefit as well." *Pharmacist, UP*
**Possibilities in future for pharmacies as a channel**	
One pharmacist reported that through the Jiyyo platform, they will attract more patients in the future. Another pharmacist mentioned that with the availability of telemedicine, they can provide complete care to rural patients right from consultation to providing medicines in one place.	"I am working here as a pharmacist for the last 11 years. I help patients with the right advice and provide them with medicines. Doctors are not there in our villages, so I joined the Jiyyo platform because, through this, I can support the patients through the entire process, right from consulting with good doctors from big cities to providing them with the medicines as prescribed." *Pharmacist, UP*

Table 17: Pharmacy Services

References

1. FIP. Beating non-communicable diseases in the community: The contribution of pharmacists. Tech. rep. 2019. URL: https://www.fip.org/files/content/publications/2019/beating-ncds-in-the-community-the-contribution-of-pharmacists.pdf.
2. Mahmood SS, Iqbal M, Hanifi SMA, Wahed T, and Bhuiya A. Are 'Village Doctors' in Bangladesh a curse or a blessing? BMC International Health and Human Rights 2010;10:18.
3. Ahmed SM, Evans TG, Standing H, and Mahmud S. Harnessing pluralism for better health in Bangladesh. The Lancet 2013;382:1746–55.
